# Supplementary material for: Distinct epigenomic patterns are associated with haploinsufficiency and predict risk genes of developmental disorders
Source: Nat Commun. 2018 May 30;9:2138. doi: 10.1038/s41467-018-04552-7 (PMC5976622; doi:10.1038/s41467-018-04552-7)
Supplement: Supplementary file 1 — Supplementary Information [file 41467_2018_4552_MOESM1_ESM.pdf]

Distinct Epigenomic Patterns Are Associated with Haploinsufficiency and Predict Risk Genes of Developmental Disorders

Han et al.

Supplementary Information

I. Supplementary Figures

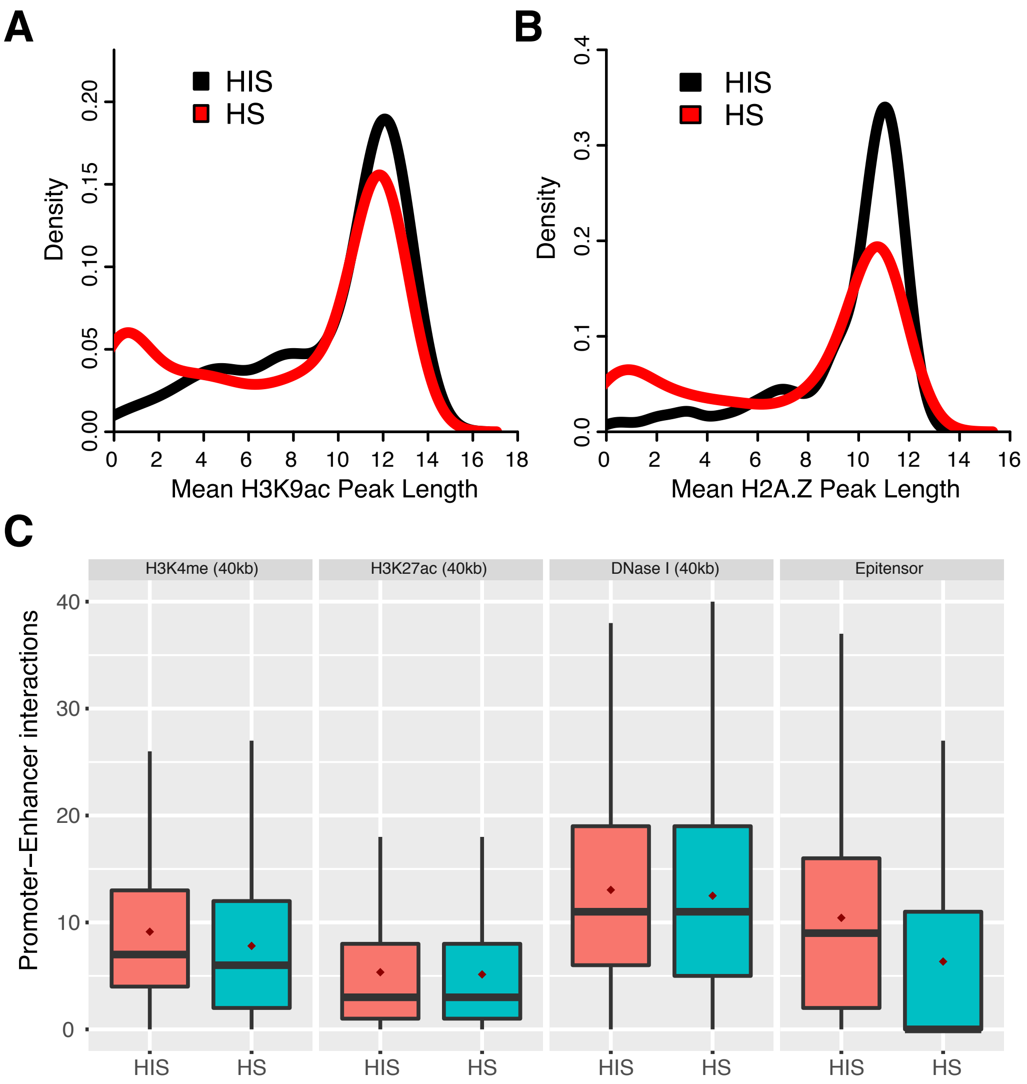

Supplementary Figure 1. The disparity of HIS and HS genes in the distribution of epigenetic features. (A-B) HIS and HS genes have different distributions of peak length from promoter features (A, H3K9ac; B, H2A.Z). (C) HIS genes have larger numbers of interacting enhancers than HS genes. When interacting enhancers were measured as the number of peaks in +/- 20kb of TSS (C, the left 3 panels), little difference between HIS and HS genes were observed. When interacting enhancers were inferred by EpiTensor (C, the rightmost panel), there is significant difference between HIS and HS genes ($p < 10$-4, permutation test of difference between medians).

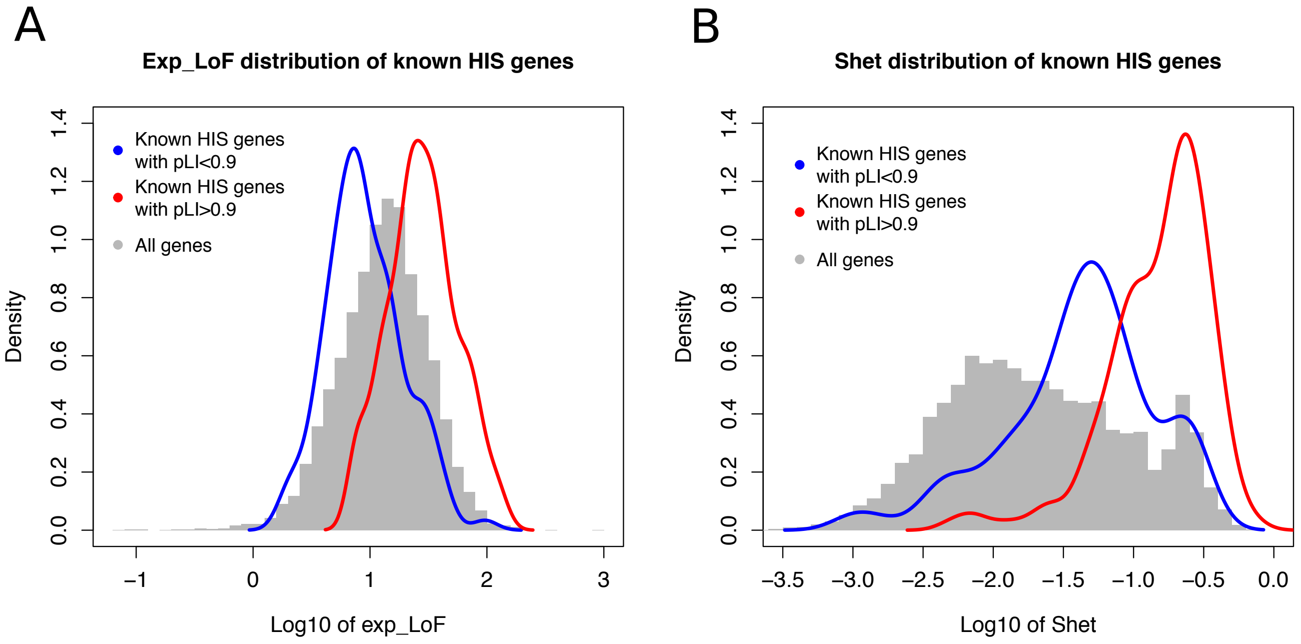

Supplementary Figure 2. Property of mutation intolerance and selection of known haploinsufficient genes used in training. The known genes are divided into two groups based on ExAC pLI scores: above (red) and below (blue) 0.9. (A) The number of expected loss of function (exp_LoF)[1] distribution of genes with pLI >0.9 or pLI<0.9. The exp_LoF value is proportional to background mutation rate, which in turn is largely determined by transcript size. Known HIS genes with pLI $<$ 0.9 have significantly smaller exp_LoF than an average gene, and the ones with pLI $>$ 0.9 have much larger exp_LoF. (B) The S_{het} (average select coefficient of heterozygous loss of function variants in a gene[2]) distribution of genes with pLI>0.9 or pLI<0.9. S_{het} values. Known HIS genes with pLI $<$ 0.9 have intermediate S_{het}: larger than than an average gene but smaller than the ones with pLI $>$ 0.9.

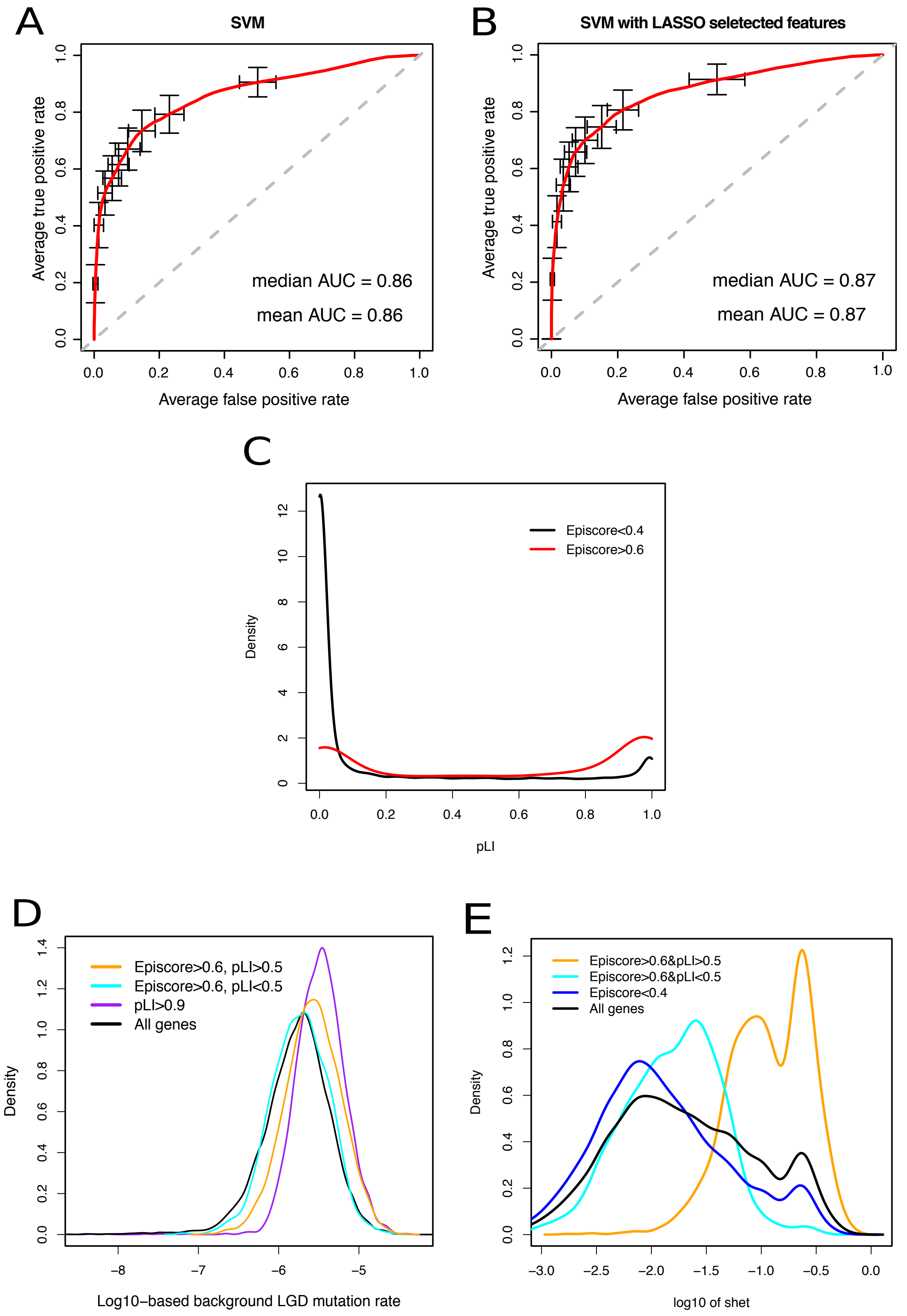

Supplementary Figure 3. Performance of various machine learning approaches and concordance of Episcore with pLI. (A-B) ROC curve of 10-fold cross-validation from applying

SVM (A) or SVM with Lasso feature selection (B) to the same epigenetic data as used in the Random Forest model. The red curve is the average of 100 randomized cross-validation runs, with error bar showing standard deviation. (C) pLI distribution of Episcore < 0.4 and Episcore >0.6 genes. The genes with Episcore > 0.6 are much more likely to have pLI values close to 1 than the genes with Episcore < 0.4, and less likely to have pLI values close to 0 than the genes with Episcore <0.4. (D) The distribution of background LGD mutation rate (log10). The genes with Episcore>0.6 and pLI < 0.5 have similar background mutation rate as an average gene, whereas the genes with pLI > 0.5 have higher background mutation rate, and the ones with pLI > 0.9 have even higher background rate. (E) The distribution of S_{het} [2]: genes with Episcore >0.6 and pLI < 0.5 have intermediate S_{het} values that are larger than an average gene and smaller than the genes with pLI>0.5. The genes with Episcore < 0.4 on average have reduced S_{het} compared to other genes.

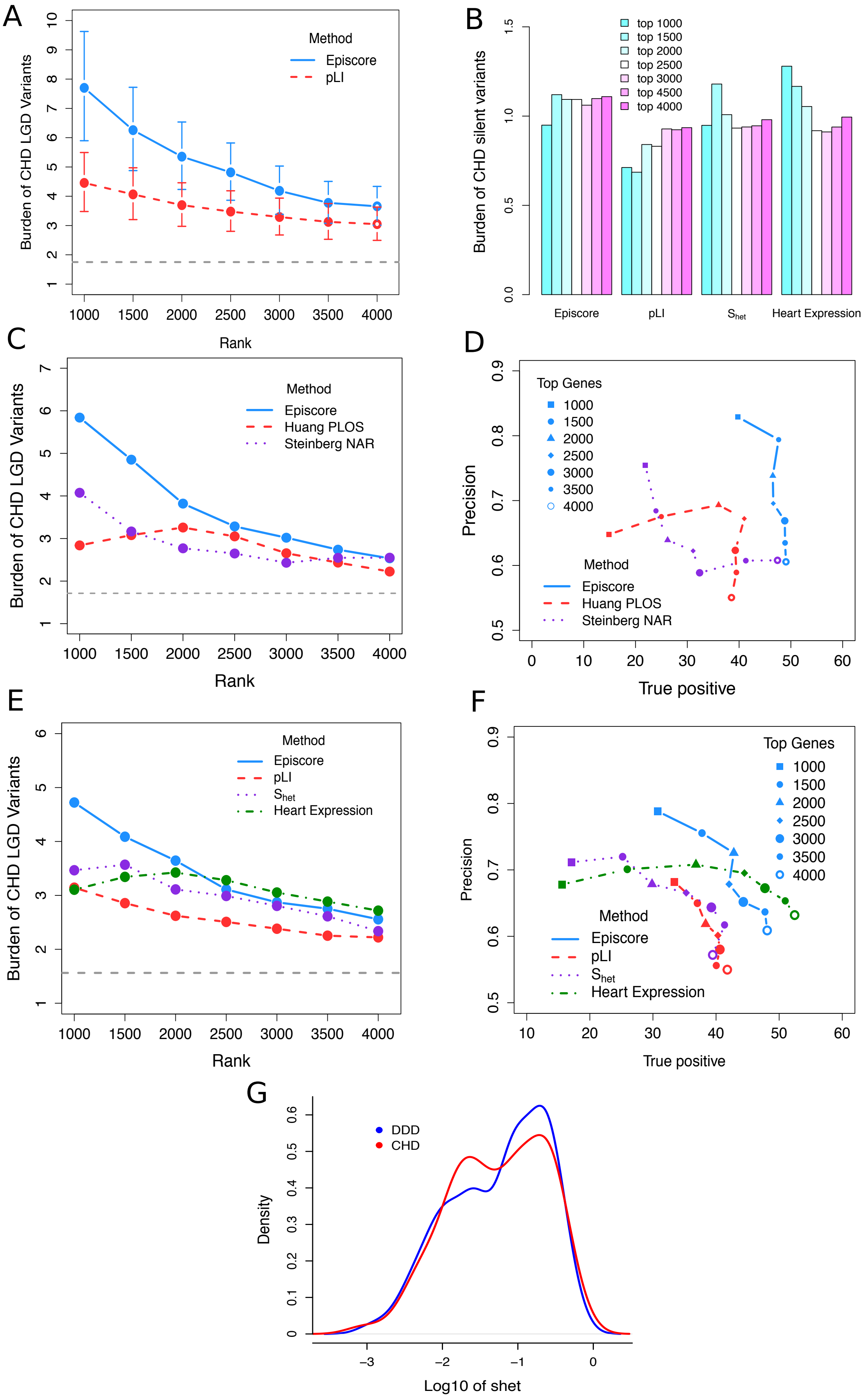
A
Burden of CHD LGD Variants
Rank
Method
Episcore
pLI
B
Burden of CHD silent variants
top 1000
top 1500
top 2000
top 2500
top 3000
top 4500
top 4000
Episcore
pLI
Shet
Heart Expression
C
Burden of CHD LGD Variants
Rank
Method
Episcore
Huang PLOS
Steinberg NAR
D
Precision
True positive
Top Genes
1000
1500
2000
2500
3000
3500
4000
Method
Episcore
Huang PLOS
Steinberg NAR
E
Burden of CHD LGD Variants
Rank
Method
Episcore
pLI
Shet
Heart Expression
F
Precision
True positive
Top Genes
1000
1500
2000
2500
3000
3500
4000
Method
Episcore
pLI
Shet
Heart Expression
G
Density
Log10 of shet
DDD
CHD

Supplementary Figure 4. Using empirical data to benchmark the performance of Episcore in variant prioritization. (A) Comparison of enrichment burden between Episcore and pLI, shown with 95% confidence intervals calculated based on Poisson distribution. (B) Enrichment of CHD silent *de novo* variants is close to 1 regardless of Episcore rank. (C-D) Comparing Episcore to prediction of haploinsufficient genes from two previous studies based on protein interaction networks [3,4], using CHD exome sequencing data. The grey dash line indicates the burden of *de novo* LGD variants acorss the genome. (E-F) Comparison of Episcore, pLI, S_{het} and heart expression level excluding known HIS genes used in training. Episcore achieves better performance than mutation intolerance-based metrics. (G) The distribution of S_{het} (log10) of genes that have LGD *de novo* mutations in DDD ID and CHD cases. Overall a larger fraction of genes with mutations in DDD ID cases have high S_{het} values, indicating the disease-causing genes are under more severe selection on average.

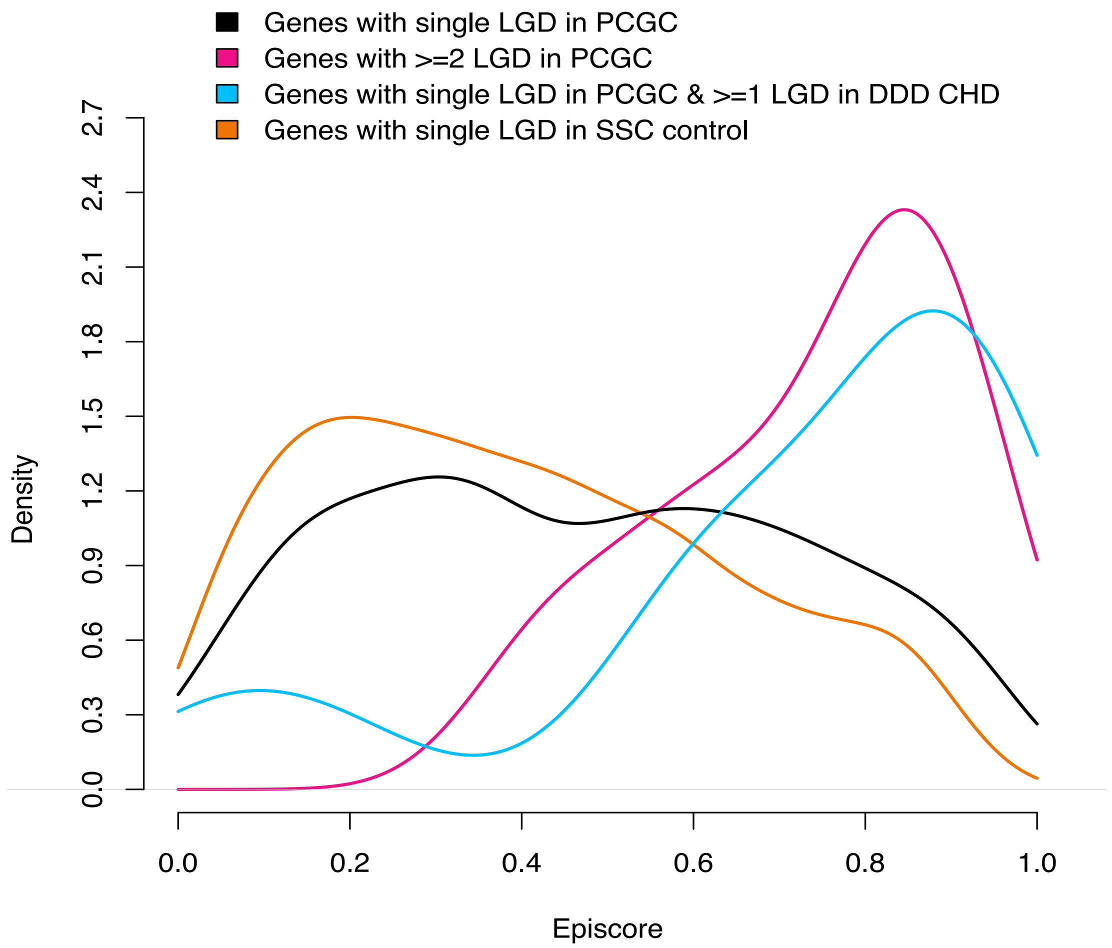

Supplementary Figure 5. Episcore distribution of genes with *de novo* LGD variants in DDD CHD cohort [5] and PCGC CHD cohort [6]. Data in an earlier version of PCGC CHD cohort [7] is depleted from DDD CHD data [5] due to duplication. The distribution of genes with single LGD variant in PCGC cohort and at least one LGD or D-mis variant in DDD CHD cohort are close to the distribution of genes with multiple LGD variants in PCGC cohort, suggesting that Episcore facilitates discovery of *de novo* risk genes with only one LGD variant. For comparison, genes with de novo single LGD variant detected from a SSC control cohort [8] have lower Episcore distribution.

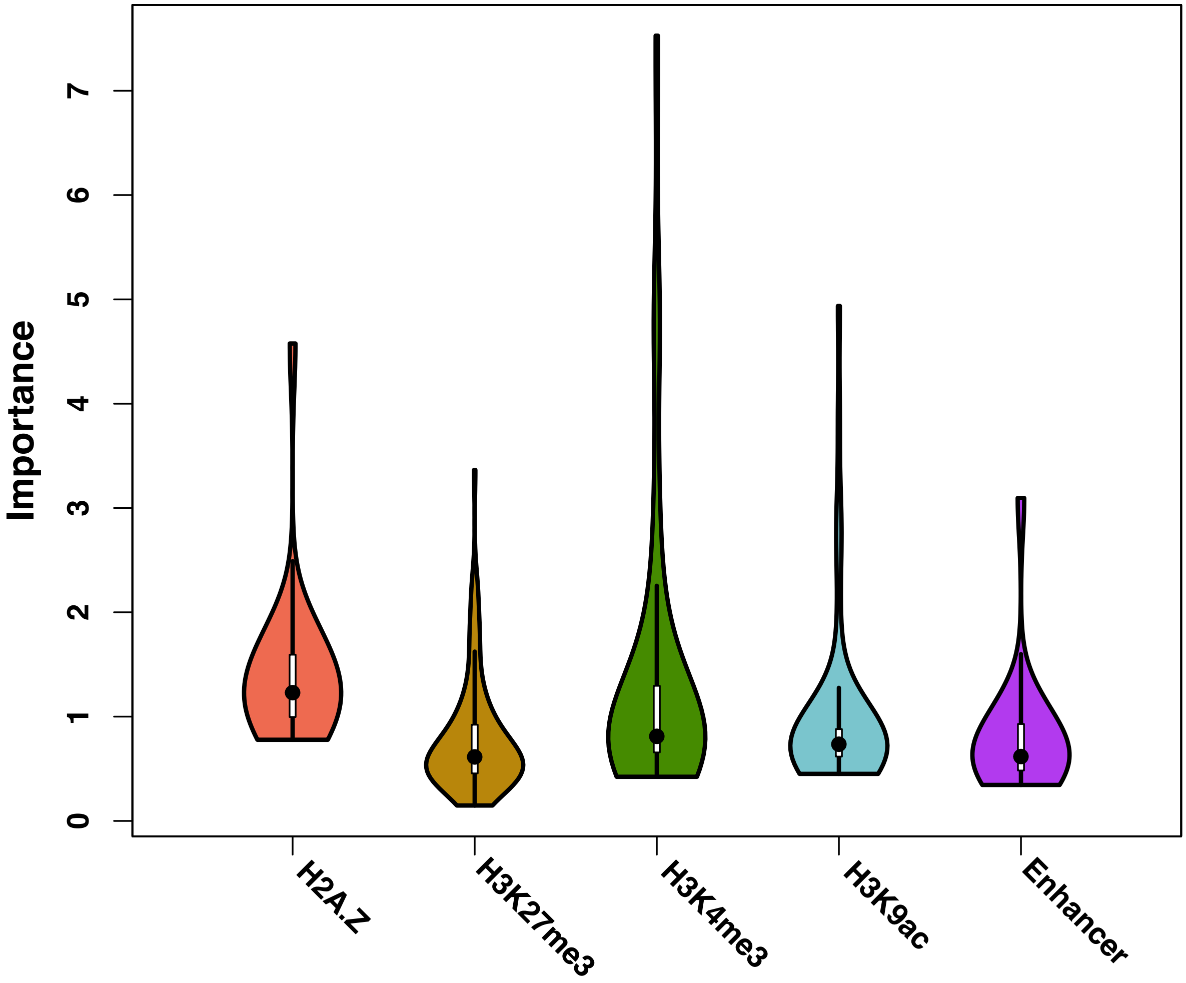

Supplementary figure 6. The importance (mean decrease of Gini index) of each feature to Episcore prediction. We obtained the importance values from the randomForest R package. Features are grouped by epigenomic molecular entities. For each group, we summarize the distribution of importance metric across cell and tissue types. Active promoter and enhancer features (H3K4me3, H3K9ac, H2A.Z, Enhancer) show higher importance than repressive promoter features (H3K27me3).

II. Supplementary Tables

Supplementary Table 1 HIS gene examples of Episcore prediction implicated in human diseases under a dominant model

Gene Symbol	Ensembl ID	Episcore	pLI	Exp LoF	ExAC LoF	Disease Relevance
PRRX1	ENSG00000116132	0.91	0.75	8.7	1	AGOTC (Donnelly et al., 2012[9])
CALM2	ENSG00000143933	0.93	0.86	6.1	0	LQT15 (Crotti et al., 2013[10]; Makita et al., 2014[11])
H3F3A	ENSG00000163041	0.94	0.69	3.6	0	PG and DIPG (Wu et al., 2012[12])
NRN1	ENSG00000124785	0.93	0.82	5.5	0	Intellectual disability (Kuipers et al., 2013[13])
HMX3	ENSG00000188620	0.91	0.77	4.6	0	Hearing loss (Miller et al., 2009[14]); Inner ear abnormalities (Sangu et al., 2016[15])
HMGB1	ENSG00000189403	0.96	0.63	7.2	1	Intellectual disability (Bartholdi et al, 2014[16])
KLLN	ENSG00000227268	0.96	NA	NA	NA	CWS4 (Bennett et al. 2010[17])
EFNA5	ENSG00000184349	0.92	0.89	6.9	0	ARC (Lin Q et al. 2014[18])
HEY2	ENSG00000135547	0.93	0.44	9.5	2	VSD and AVSD (Reamon-Buettner et al. 2006[19])
ASF1A	ENSG00000111875	0.88	0.14	5.5	2	EA (Giannakou et al. 2017[20])
CDK13	ENSG00000065883	0.90	0.75	43.9	9	CHD (Sifrim et al. 2016[5]; Hamilton et al. 2018[21])
PRDM6	ENSG00000061455	0.91	NA	NA	NA	PDA3 (Li et al. 2016[22])
LMO4	ENSG00000143013	0.88	0.82	5.3	0	Breast cancer (Sutherland et al. 2003[23])
POU3F2	ENSG00000184486	0.89	NA	NA	NA	HD (Costa et al. 2006[24])
MKX	ENSG00000150051	0.91	0.88	11.1	1	Cryptorchidism (Mroczkowski et al. 2014[25])
HAND2	ENSG00000164107	0.87	0.35	4.3	1	CHD (Sun et al. 2016[26])

References

1. Lek, M. *et al.* Analysis of protein-coding genetic variation in 60,706 humans. *Nature* **536**, 285-91 (2016).
2. Cassa, C.A. *et al.* Estimating the selective effects of heterozygous protein-truncating variants from human exome data. *Nat Genet* **49**, 806-810 (2017).
3. Huang, N., Lee, I., Marcotte, E.M. & Hurles, M.E. Characterising and predicting haploinsufficiency in the human genome. *PLoS Genet* **6**, e1001154 (2010).
4. Steinberg, J., Honti, F., Meader, S. & Webber, C. Haploinsufficiency predictions without study bias. *Nucleic Acids Res* **43**, e101 (2015).
5. Sifrim, A. *et al.* Distinct genetic architectures for syndromic and nonsyndromic congenital heart defects identified by exome sequencing. *Nat Genet* **48**, 1060-5 (2016).
6. Jin, S.C. *et al.* Contribution of rare inherited and de novo variants in 2,871 congenital heart disease probands. *Nat Genet* (2017).
7. Zaidi, S. *et al.* De novo mutations in histone-modifying genes in congenital heart disease. *Nature* **498**, 220-3 (2013).
8. Krumm, N., O'Roak, B.J., Shendure, J. & Eichler, E.E. A de novo convergence of autism genetics and molecular neuroscience. *Trends Neurosci* **37**, 95-105 (2014).
9. Donnelly, M., Todd, E., Wheeler, M., Winn, V.D. & Kamnasaran, D. Prenatal diagnosis and identification of heterozygous frameshift mutation in PRRX1 in an infant with agnathia-otocephaly. *Prenat Diagn* **32**, 903-5 (2012).
10. Crotti, L. *et al.* Calmodulin mutations associated with recurrent cardiac arrest in infants. *Circulation* **127**, 1009-17 (2013).
11. Makita, N. *et al.* Novel calmodulin mutations associated with congenital arrhythmia susceptibility. *Circ Cardiovasc Genet* **7**, 466-74 (2014).
12. Wu, G. *et al.* Somatic histone H3 alterations in pediatric diffuse intrinsic pontine gliomas and non-brainstem glioblastomas. *Nat Genet* **44**, 251-3 (2012).
13. Kuipers, B.C. *et al.* Two patients with intellectual disability, overlapping facial features, and overlapping deletions in 6p25.1p24.3. *Clin Dysmorphol* **22**, 18-21 (2013).
14. Miller, N.D. *et al.* Molecular (SNP) Analyses of Overlapping Hemizygous Deletions of 10q25.3 to 10qter in Four Patients: Evidence for HMX2 and HMX3 as Candidate Genes in Hearing and Vestibular Function. *American Journal of Medical Genetics Part A* **149a**, 669-680 (2009).

15. Sangu, N. *et al.* A de novo microdeletion in a patient with inner ear abnormalities suggests that the 10q26.13 region contains the responsible gene. *Hum Genome Var* **3**, 16008 (2016).
16. Bartholdi, D. *et al.* A newly recognized 13q12.3 microdeletion syndrome characterized by intellectual disability, microcephaly, and eczema/atopic dermatitis encompassing the HMGB1 and KATNAL1 genes. *Am J Med Genet A* **164A**, 1277-83 (2014).
17. Bennett, K.L., Mester, J. & Eng, C. Germline epigenetic regulation of KILLIN in Cowden and Cowden-like syndrome. *JAMA* **304**, 2724-31 (2010).
18. Lin, Q., Zhou, N., Zhang, N. & Qi, Y. Mutational screening of EFNA5 in Chinese age-related cataract patients. *Ophthalmic Res* **52**, 124-9 (2014).
19. Reamon-Buettner, S.M. & Borlak, J. HEY2 mutations in malformed hearts. *Hum Mutat* **27**, 118 (2006).
20. Giannakou, A. *et al.* Copy number variants in Ebstein anomaly. *PLoS One* **12**, e0188168 (2017).
21. Hamilton, M.J. *et al.* Heterozygous mutations affecting the protein kinase domain of CDK13 cause a syndromic form of developmental delay and intellectual disability. *J Med Genet* **55**, 28-38 (2018).
22. Li, N. *et al.* Mutations in the Histone Modifier PRDM6 Are Associated with Isolated Nonsyndromic Patent Ductus Arteriosus. *Am J Hum Genet* **99**, 1000 (2016).
23. Sutherland, K.D. *et al.* Mutational analysis of the LMO4 gene, encoding a BRCA1-interacting protein, in breast carcinomas. *Int J Cancer* **107**, 155-8 (2003).
24. Costa, M.D. *et al.* Exclusion of mutations in the PRNP, JPH3, TBP, ATN1, CREBBP, POU3F2 and FTL genes as a cause of disease in Portuguese patients with a Huntington-like phenotype. *Journal of Human Genetics* **51**, 645-651 (2006).
25. Mroczkowski, H.J., Arnold, G., Schneck, F.X., Rajkovic, A. & Yatsenko, S.A. Interstitial 10p11.23-p12.1 Microdeletions Associated with Developmental Delay, Craniofacial Abnormalities, and Cryptorchidism. *American Journal of Medical Genetics Part A* **164**, 2623-2626 (2014).
26. Sun, Y.M. *et al.* A HAND2 Loss-of-Function Mutation Causes Familial Ventricular Septal Defect and Pulmonary Stenosis. *G3 (Bethesda)* **6**, 987-92 (2016).
