# Supplementary material for: Distinct epigenomic patterns are associated with haploinsufficiency and predict risk genes of developmental disorders
Source: Nat Commun. 2018 May 30;9:2138. doi: 10.1038/s41467-018-04552-7 (PMC5976622; doi:10.1038/s41467-018-04552-7)
Supplement: Supplementary file 2 — Description of Additional Supplementary Files [file 41467_2018_4552_MOESM2_ESM.pdf]

**Description of Additional Supplementary Files**

File Name: Supplementary Data 1
Description: Genes used for random forest model training.

File Name: Supplementary Data 2
Description: Human diseases and biological pathways implicated with known HIS Genes.

File Name: Supplementary Data 3
Description: Predicted Episcore of all protein-coding genes.
